# Influence of lifestyle factors on long-term sickness absence among female healthcare workers: a prospective cohort study

**DOI:** 10.1186/1471-2458-14-1084

**Published:** 2014-10-18

**Authors:** Helle Gram Quist, Birthe L Thomsen, Ulla Christensen, Thomas Clausen, Andreas Holtermann, Jakob B Bjorner, Lars L Andersen

**Affiliations:** National Research Centre for the Working Environment, Lerso Parkallé 105, 2100 Copenhagen, Denmark; Department of Public Health, University of Copenhagen, Section of Social Medicine, Oster Farimagsgade 5, 1014 Copenhagen, Denmark; QualityMetric/OptumInsight, 24 Albion Road, 02865 Lincoln, RI USA

**Keywords:** Long-term sickness absence, Lifestyle, Smoking, Body mass index, Physical activity, Cohort study

## Abstract

**Background:**

While previous research has indicated that unhealthy lifestyle is associated with sickness absence, this association may be confounded by occupational class. To avoid this potential confounding, we examined the association between lifestyle factors (smoking, leisure-time physical activity and body mass index) and the occurrence of long-term sickness absence (LTSA; more than three consecutive weeks of registered sickness absence) within a cohort of female health care workers.

**Methods:**

A total of 7401 employees filled out a questionnaire about their health behaviour and work environment. Subsequently, they were followed for 12 months in a national register on social transfer payments (DREAM register). Cox’s regression analyses, applied to grouped survival data, were used to estimate the prospective association between these lifestyle factors and LTSA.

**Results:**

We found significant associations between all three lifestyle factors and risk of LTSA. The strongest lifestyle factor was current smoking, which increased the risk of LTSA by 35% (95% CI: 1.17-1.54) compared to non- smokers. For body mass index, the risk of LTSA increased with the distance away from 18.5 kg/m^2^ in either direction (below 18.5 kg/m^2^: HR: 1.32 per kg/m^2^; 95% CI. 1.06-1.66; above 18.5 kg/m^2^: HR: 1.04 per kg/m^2^; 95% CI: 1.03-1.05). In other words, the more underweight or overweight the women were, the higher the risk of LTSA. A dose–response relationship was found between LTSA and leisure-time physical activity (trend test p-value = 0.01), so that increasing physical activity results in decreasing risk of LTSA.

**Conclusion:**

In female healthcare workers, an unhealthy lifestyle (too high/ too low body mass index, smoking, and low physical activity) is associated with higher risk of LTSA.

## Background

Sickness absence, and in particular, the reduction of sickness absence, has been on the political agenda in Denmark for years. The healthcare sector ranks particular high in sickness absenteeism [1]. To reduce the costs of sickness absence for the society, regulations on sick leave have become more restricted in the past years [2]. Further, worksites have increased their focus on general well-being and health promotion for the employees in an attempt to reduce sickness absence and improve work attendance. Sickness absence express a complex relationship between health and work characteristics [3] and is caused by a variety of factors, including occupational injuries and work related factors, such as physical demands, perceived exertion and psychosocial exposures [4–7]. However, research also indicates that sickness absence, and affiliation with the labour market in general, are influenced by personal health behaviours [4, 6, 8–10]. Sickness absence can be seen as an early indicator of illness and has been suggested as a measure of health and functioning in working populations [11]. A Danish research study has found that the longer duration of sickness absence the higher the risk of future disability pension, indicating that long-term sickness absence (LTSA) is also a predictor of expulsion from the labour marked [12].

Previous research has found associations between sickness absence and smoking [8, 13–15], obesity [13–15] and leisure-time physical activity [16–18]. In general, it has been found that smoking, lack of moderate and/or vigorous leisure-time physical activity, and obesity increases the risk of sickness absence. Also, a study on the association between body mass index (BMI) and mortality found that the strongest association was for those with low BMI [19]. Together, this could potentially indicate a v-shaped relationship between BMI and LTSA as well. However, studies on the association between underweight and sickness absence are rather uncommon in the literature and the results are inconsistent [20].

It is well documented that there exist a social gradient in lifestyle. Unhealthy lifestyles, such as smoking, lack of physical activity, and poor dietary habits are associated with socioeconomic status (e.g. educational level, affiliation with the labour market and income) [21, 22]. Individuals with short educational background, and those standing outside the labour market, generally have more unfavourable lifestyles than those with a longer education [22]. Other significant predictors of sickness absence are occupational class and working conditions [9, 10, 15, 23, 24]. These may therefore be potential confounders in the association between lifestyle and sickness absence. As differences exist among different occupational groups, it is important to control for social class – e.g. by stratification or restriction of the sample. Therefore, we have studied the association between lifestyle and sickness absence in a homogenous group of health care workers. Previously mentioned studies [8, 13, 14, 16–18] controlled for social class either by socioeconomic position, occupational class or by restriction of the sample. The approach of examining the association among health care workers only, helps us circumvent the issue of confounding by social class to a large extent. Furthermore, research indicates that women have higher rates of sickness absence than men [25]. The work environment also seem to have different effects of sickness absence for men and women [26, 27]. This study is limited to women.

The aim of this study was to investigate the prospective association between lifestyle factors (smoking, BMI and leisure-time physical activity) and the risk of LTSA in a female cohort of health care workers, with and without adjustment for work related factors.

## Methods

The present study is based on survey data collected by postal questionnaires among female employees in the eldercare sector in Denmark. Data on long-term sickness absence was obtained by linkage to the Danish Register for Evaluation of Marginalization (DREAM), which is a register on social transfer payments [28]. The survey was conducted in the fall of 2004 and spring of 2005 and aimed at examining the health and working conditions of health care workers. Of the 65 municipalities invited to participate, 36 enrolled in the study. A total of 9,949 employees (78% of the total 12,746 employees) chose to participate. Analyses indicated that the participation rate was lowest among men, younger employees, and among service personnel [29]. There was a slight overrepresentation of small municipalities in the study, and although the participating municipalities were not representative in terms of geographically location, they were representative in terms of demographics, the labour market, service-level within the healthcare area and socioeconomic wealth in the municipality.

We excluded men (n = 429) and employees in management or service production (e.g. kitchen staff, janitors, secretaries etc.; n = 1586) from the analyses. It was required that all respondents were healthy (not receiving sickness benefits) at baseline and were associated with the work force. Thus, we excluded a total of 419 respondents with current sickness absence at baseline and 114 for having an uncertain affiliation with the work force at baseline (e.g. unemployment, maternity leave, starting allowance, or rehabilitation benefits).

As a result, the target population consisted of 7401 female employees in health-related care services functions (2548 respondents were excluded in total). Due to the exclusion of men and those in management and service functions, the lower response rate among these employees is not considered an issue for the analyses pertaining to this study. The respondents were subsequently identified in the DREAM register and followed for one year after completion of the questionnaire. The study has been approved by the Danish Data Protection Agency. According to Danish law, questionnaire research does not require approval by ethic committees or informed consent.

### Outcome variable: long-term sickness absence

We wanted to study LTSA using an objective register based measurement to avoid response bias on the outcome. In Denmark, all social benefits, including sickness absence benefits, are registered in the DREAM register [28]. LTSA from the DREAM register [28] was linked to survey data via the unique Danish personal identification number, which is a ten-digit number given to all Danish citizens at birth. The DREAM register contains weekly information about all public transfer payments and includes everyone who has received social benefits or other types of transfer income since 1991. Sickness absence compensation is given to the employer by applying for a refund from the government. At the time of the study such a refund was generally given after 13 calendar days of sickness absence (though some employers are entitled to a refund from the beginning of the sick leave). The employer is required to pay for the initial period, which is referred to as the “employer period”. As sickness absence is registered per *whole* week in DREAM (and not by single days), it was necessary to choose three weeks of registred absence as cut-point in order to reflect the employer period. The validity of the sickness absence data in DREAM is considered high [30, 31].

### Lifestyle factors

Three lifestyle factors were assessed in this study: smoking, BMI and leisure-time physical activity. Smoking was categorized into current smoker, former smoker, and never smoker. Never smokers served as the reference group. BMI was calculated based on the respondents’ self-reported information on height and weight (BMI = kg/m^2^). Leisure-time physical activity was assessed with a single question: “Which description most precisely covers your pattern of physical activity at leisure time during the last 12 months?” Four response categories (ranging from low to high duration and intensity) were utilized: (i) Mainly sedentary or light physical activity for less than 2 hours per week (e.g. you read, watch television, go to the cinema); (ii) Light physical activity for 2–4 hours per week (e.g. you go for a walk, light gardening, light physical exercise); (iii) Light physical activity for more than 4 hours per week or vigorous physical exercise for 2–4 hours per week (e.g. fast jogging or cycling, heavy gardening, exercise where you are sweating and breathing heavily); (iv); Vigorous physical exercise for more than 4 hours per week or taking part in regular competitive sports several times a week [1, 32]. In the tables, these are referred to as sedentary, light, moderate (reference group) and vigorous leisure-time physical activity. Moderately active respondents were presented as the reference group as they constituted the largest group (49%).

### Confounders

The potential confounders in this study included age, tenure (years working as health care worker), occupational type, physical workload and four indicators of psychosocial work conditions and previous LTSA. The assessment of physical workload was based on the Hollmann Index questionnaire asking about body postures and weight lifted during the workday (scale 0–62; 62 represents the highest degree of physical demands) [33]. The psychosocial work environment was assessed with four dimensions from the Copenhagen Psychosocial Questionnaire (COPSOQ): (i) emotional demands (3 items, e.g. “Do you get emotionally involved in your work”), (ii) role conflicts (4 items, e.g. “Are contradictory demands placed at your work”), (iii) influence at work (4 items, e.g. “Do you have a say in choosing who you work with”) and (iiii) quality of leadership (4 items, e.g. “To what extent would you say that your immediate superior is good at solving conflicts”) [34, 35]. These particular psychosocial work factors have previously been found to be significantly associated with lifestyle [36, 37] and with the risk of LTSA among nurses within the Danish eldercare services [24]. The psychosocial factors were included separately in the model. Previous LTSA (within one year prior to completing the questionnaire) was included as a confounder in Model 4.

#### Statistical analyses

To analyse whether, and to what extent, lifestyle risk factors predicted the onset of LTSA during the first year of follow up, we used Cox’s proportional hazards model applied to grouped survival data [38]. We used calendar time as the time scale, with delayed entry at the time of responding to the questionnaire. Hazard ratios (HR) and 95% confidence intervals (95% CI) were calculated for each lifestyle risk factor.

Smoking status and leisure-time physical activity were assessed as categorical variables, while

BMI and all quantitative confounders were modelled as linear splines. We used linear splines to model the association between the variables and LTSA since using this flexible model avoids unnecessary categorization while not assuming a simple linear relationship [39]. A single knot for BMI was chosen at 18.5 kg/m^2^, which corresponds to the beginning of being normal weight of the standardized BMI categories. The adequacy of having only one knot at 18.5 kg/m^2^ was evaluated in a model using linear splines with knots according to the standard categorization of BMI (18.5, 25, and 30 kg/m^2^). For all models presented in this paper, additional knots at 25 and 30 kg/m^2^ gave no significant improvement (all p-values >0.93).

Knots for age were chosen at five-year intervals (25, 30, 35, 40, 45, 50, 55, 60 years of age), while knots for tenure were chosen at five and ten years of work experience (<5 years, between five and ten years, >10 years). The remaining confounders had the knots chosen so that approximately one-third of the women with incident LTSA were included in each interval.

In Model 1 we assessed the separate effect of each lifestyle factors on LTSA while adjusting for age. In our main model (Model 2), we examined the effects of the combined lifestyle risk factors while adjusting for age. In Model 3, we additionally adjusted for the following work-related factors: tenure, occupational type (care helper, care assistant, registered nurse etc.), physical work demands and psychosocial work dimensions. In the final model (Model 4), we also adjusted for previous long-term sickness absence (in the year prior to completion of the questionnaire).

Time since completion of the questionnaire was not included in the model, since filling out the questionnaire is unlikely to affect the incidence rate of LTSA. Each respondent was included in the analyses from completion of the questionnaire and until the occurrence of LTSA, retirement, death, emigration, or end of 12 month follow-up – whichever came first. The estimation method was maximum likelihood and the statistical analyses were performed with the SAS Proc Genmod procedure, version 9.2 (SAS Institute).

## Results

Baseline characteristics of the female health-care workers are presented in Table 1.

**Table 1:** **Descriptive statistics of the population**

	Long-term Sickness Absence		
Variable	N	Mean (2.5-97.5 percentile)	Event (%)
*Baseline 2005 (n = 7401)*			
Age	7401	45.2 (24–60)	
Seniority (years working as health care worker)^a^	7306	9.0 (1–28)	
Occupational type:			
Care helpers	4595		824 (11.2)
Care assistants	1304		198 (2.7)
Unskilled care helpers	162		24 (0.3)
Registered nurses	936		106 (1.4)
Therapists and activity workers	404		46 (0.6)
Physical activity^b^:			
Sedentary	305		60 (19.7)
Light activity	3028		518 (17.2)
Moderate activity	3604		555 (15.4)
Hard activity	341		40 (11.8)
Smoking status^c^:			
Never smoker	2817		395 (14.1)
Ex-smoker	1778		274 (15.5)
Current smoker	2724		511 (18.8)
BMI (kg/m^2^)^d^	7066	24.9 (18.7 - 35.6)	
BMI category^d^:			
Underweight (<18.5 kg/m^2^)	140		21 (15.1)
Normal weight (18.5-24.9 kg/m^2^)	4021		573 (14.3)
Overweight (25–29.9 kg/m^2^)	2059		357 (17.4)
Obese (>30 kg/m^2^)	846		184 (21.9)
Previous long-term sickness absence:			
Yes	1045		293 (28.1)
No	6356		905 (14.3)

^a^95 missing responses.

^b^123 missing responses.

^c^82 missing responses.

^d^335 missing responses.

A total of 1198 people (16.2%) experienced LTSA within one year after completing the questionnaire. On average the respondents were 45.2 years old and had worked as health care workers for 9 years. Approximately 82% were care helpers and assistants, while almost 13% were registered nurses and 2% were therapists. On average the respondents had a BMI of 24.9 and approximately every other person (46%) indicated that they had a light or sedentary leisure-time physical activity level.

After adjusting for age, the risk of LTSA was significantly increased by smoking and BMI (Table 2, Model 2). Being a current smoker was associated with an increased risk of LTSA of 34% (95% CI: 1.17-1.54) compared to non-smokers (the reference group). Being a former smoker was not associated with a higher risk of LTSA (HR: 1.05; 95% CI: 0.89-1.23). In all four models, the association with leisure-time physical activity could be reduced to a general trend (all p-values >0.5756) and a higher physical activity was associated with a lower risk of LTSA (all trend test p-values < 0.0140; results not shown). In other words, we found a dose–response relationship between leisure-time physical activity and LTSA, in that sedentary physical activity increased the risk of LTSA, while high physical activity protected against LTSA.

**Table 2:** **Hazard Ratios and confidence intervals for onset of long-term sickness absence during 12-months of follow-up**

	N	Model 1		Model 2		Model 3		Model 4	
Variable		HR (95% CI)	P-value*	HR (95% CI)	P-value*	HR (95% CI)	P-value*	HR (95% CI)	P-value*
Leisure-time physical activity			**0.0086**		0.0844		**0.0420**		0.0602
Sedentary	305	1.34 (1.02-1.75)		1.28 (0.97-1.68)		1.34 (1.02-1.77)		1.35 (1.02-1.78)	
Light	3028	1.13 (1.00-1.28)		1.09 (0.96-1.23)		1.09 (0.96-1.24)		1.08 (0.95-1.22)	
Moderate	3604	1.00 -		1.00 -		1.00 -		1.00 -	
Vigorous	341	0.77 (0.56-1.06)		0.81 (0.58-1.12)		0.79 (0.56-1.10)		0.80 (0.57-1.13)	
*Missings*	*123*								
Smoking			**<.0001**		**<.0001**		**0.0045**		**0.0066**
Never smoker	2817	1.00 -		1.00 -		1.00 -		1.00 -	
Former smoker	1778	1.09 (0.93-1.27)		1.05 (0.89-1.23)		1.01 (0.86-1.19)		1.00 (0.84-1.18)	
Current smoker	2724	1.33 (1.17-1.52)		1.35 (1.17-1.54)		1.24 (1.08-1.44)		1.23 (1.06-1.42)	
*Missings*	*82*								
BMI			**<.0001**		**<.0001**		**<.0001**		**<.0001**
1 point lower BMI below 18.5	140	1.35 (1.08-1.69)		1.32 (1.06-1.66)		1.31 (1.04-1.66)		1.33 (1.05-1.69)	
1 point higher BMI above 18.5	6926	1.04 (1.03-1.05)		1.04 (1.03-1.06)		1.04 (1.02-1.05)		1.03 (1.02-1.05)	
*Missings*	*335*								
LTSA									**<.0001**
PreviousLTSA	7401	.	.	.	.	.	.	2.02 (1.75-2.33)	

Model 1: separate analyses, adjusted for age.

Model 2: combined lifestyle factors, adjusted for age.

Model 3: combined lifestyle factors, adjusted for age, tenure, physical and psychosocial work factors, occupational type.

Model 4: combined lifestyle factors, adjusted for age, tenure, physical and psychosocial work factors, occupational type and previous long-term sickness absence.

*P-values concern an overall test for the variable in question.

Significant results are presented in boldface.

Regarding BMI, we found that the risk of LTSA was higher the further away BMI was from 18.5 kg/m^2^ (in either direction). For individuals with a BMI below 18.5 kg/m^2^ the risk of LTSA increased by 32% per 1 kg/m^2^. For individuals with a BMI above 18.5 kg/m^2^ the risk of LTSA increased by 4% per 1 kg/m^2^. In other words, the more underweight or overweight a person is the higher the risk of LTSA. Additional calculations indicate that two people with BMI of, respectively, 16.5 kg/m^2^ and 32 kg/m^2^ have the same increased risk of LTSA; a relative risk of 1.74 compared to women with a BMI of 18.5 kg/m^2^ and the same status on other risk factors (results not shown). We adjusted for a range of confounders (age, tenure, occupational type, physical workload, four measures of psychosocial work environment, and previous long-term sickness absence), but this adjustment did not alter the significant associations between the lifestyle risk-factors and LTSA, nor did it change the estimates in any sizeable way (Table 2, Model 3 and 4).

## Discussion

### Summary of findings

This study investigated the association between lifestyle and LTSA among female health care workers in Denmark. LTSA was significantly associated with all three health-related behaviours (smoking, physical activity and BMI). In line with previous studies [13, 15, 18, 20, 40, 41], we found that an unhealthy lifestyle, defined in terms of smoking, being physically inactive and having a high BMI, increased the risk of LTSA. In addition, we found that people with a BMI below 18.5 kg/m^2^ have increased risk of LTSA with a higher risk for lower scores. This area is somewhat unstudied and results have previously been inconsistent. Neovius and collegues [40] found that underweight individuals had increased risk for short episodes of sickness absence, but decreased risk of long-term episodes, while Parkes [42] found a J-shaped relationship between BMI and sick-leave frequency, showing that underweight, overweight and obese individuals had increased risk of sick-leave compared to normal weight individuals. Our findings add to this rather overlooked area. In fact our results indicate that for women, being underweight is equally dangerous as being obese with regards to LTSA. However, in a society that puts such pressure on women for being skinny, the detrimental effects of being underweight are often neglected and ignored.

Smoking compared to non-smoking showed increased risk of LTSA of 35%. This is in line with previous findings [13–15, 43, 44]. However, former smokers were not at increased risk of LTSA in our study, which contradicts findings from previous research [8, 15, 43]. With regards to leisure-time physical activity, we found a significant association with LTSA, supporting the notion that an inactive lifestyle increases the risk of LTSA. We found a dose–response pattern where vigorous physical activity seems to have a protective effect on LTSA. Other studies have also reported that vigorous physical activity during leisure-time is associated with reduced sickness absence [16–18, 45]. Holtermann and colleagues investigated the effects of both occupational and leisure-time physical activity on LTSA in the general Danish population [46]. They also found that leisure-time physical activity decreased the risk of LTSA; however, occupational physical activity increased the risk of LTSA, indicating opposing effects of occupational and leisure-time physical activity [46].

The association between LTSA and lifestyle cannot be explained by confounding from the measured covariates; even with adjustment for working conditions, previous sickness absence and occupational type, all three lifestyle factors were strongly associated with LTSA. In other words, lifestyle factors are independent risk factors that influence sickness absence regardless of work environment.

### Strength and limitations

A potential advantage of our study is the robust study design. We relied on register data, which we linked to the survey data, giving us valid measures on sickness absence. In other words, we did not have to rely on self-reported sickness data which are prone to recall bias. Furthermore, the DREAM register covers every person who has received social transfer payment, which eliminates dropout. Another strength of our study is that we investigated long-term sickness absence rather than short-term sickness absence. Causes of long- and short-term sickness absence can be very different; short spells of sickness absence can potentially reflect other factors than actual sickness (such as lack of motivation, truanting, pregnancy or the burden of family obligations [47, 48]), while long-term sickness absence mostly requires medical confirmation from a doctor and is a predictor of expulsion from the labour marked in itself [12]. Investigating the associations among healthcare workers only is a strength of this study as it allows us to say something concrete about this particular occupational groups. However, the associations seen in this study cannot be generalized to e.g. men, other occupational sectors or other countries. Further studies are needed to evaluate the association between LTSA and lifestyle in other working populations. Restricting our population to a homogenous occupational group limited the social gradient as a source of confounding to a large extent. Thus, the study shows that the association between LTSA and lifestyle factors goes beyond simple confounding by social class. A limitation of our study was the use of self-reported data on all lifestyle variables; the use of self-reported data can cause bias as e.g. self-reported height and weight are often over-and underestimated, respectively [49, 50]. The study relied on data originating from 2005 and results may not be directly transferable to today because of changes in sickness absence benefits. However, the changes have only affected the rules for payment/reimbursement of sickness benefits to the employer. Since LTSA is largely reflecting chronic disease [51] it is probably less affected by changes in benefits than short time sickness absence. For these reasons, we assess that the association between lifestyle and LTSA is robust to changes in sickness benefits. Finally, it should be noted that only 140 participants had a BMI below 18.5 kg/m^2^, which could be relatively close to some floor value of BMI needed for survival (flooring effect). Thus, this could potentially explain the stronger effect per kg/m^2^ among the underweight individuals.

### Implications

The identified associations between lifestyle and LTSA suggest some workplace benefits of promoting healthy lifestyles. The cost of sickness absence (due to lost productivity, health-care costs etc.) may serve as a motivation for companies to provide employees with health-promoting initiatives, such as exercise rooms, changes in the available food and beverages, or offering weight loss- and smoking cessation programs. Thus, improving employee health through worksite changes and initiatives can potentially lead to a reduction in sickness absence. Smoking bans, food- and alcohol regulations, creating visible stairways and adding exercise rooms are worksite structural changes that can endorse a healthier choice. In a comprehensive review, Mozaffarian and colleagues systematically reviewed evidence-based population-based strategies that has been proven efficient in helping lifestyle changes [52]. They found that certain workplace interventions and programmes, such as changes in available food and beverages, nutritional education, stress management, and smoking restrictions did improved dietary habits and reduced the use of tobacco [52]. However, many of the recent reviews, including the before-mentioned, indicate the need for more well-designed studies, in particularly with regards to physical activity as the effectiveness of these are limited and inconclusive [52, 53]. Similarly, a recent meta-analysis on the effectiveness of workplace health promotion programs by Rongen et al. [54] concluded that the overall effect was small. Rongen and colleagues [54] found that it was not only the intervention itself, but also the underlying components such as the study population, study characteristics and quality of the methodology, that were key components for the effectiveness. Workplace health promotion programs were more effective among younger populations, when interventions included weekly contacts and when the control group received no health promotion [54].

## Conclusion

This study adds knowledge about the association between lifestyle and sickness absence, and addresses the somewhat overlooked relationship between underweight and sickness absence. We found that unhealthy behaviours (smoking, extreme BMI, and low physical activity) are predictive of LTSA among female health care workers. Further, it points to the potential of reducing long-term sickness absence if improvements are made regarding personal health behaviours, such as quitting smoking, increased physical activity and obtaining/maintaining a BMI in the normal range. In order to reduce sickness absence, which would provide important societal and personal gains, the society, community and worksites must, in collaboration with each other, encourage, support and plan for health-promoting programmes aimed at improving healthy lifestyles in addition to ensuring a safe work environment.

## Abbreviations

LTSA: Long-term sickness absence
BMI: Body mass index
DREAM: Danish register for evaluation of marginalization
COPSOQ: Copenhagen psychosocial questionnaire
HR: Hazard ratios
CI: Confidence interval.
